# Evaluation of different temporal periods between hormone-induced ovulation attempts in the female Fowler’s toad *Anaxyrus fowleri*

**DOI:** 10.1093/conphys/coz113

**Published:** 2020-01-09

**Authors:** Emmet L Guy, Michelle W Martin, Andrew J Kouba, Judith A Cole, Carrie K Kouba

**Affiliations:** Department of Biochemistry, Molecular Biology, Entomology, and Plant Pathology, Mississippi State University, Starkville, MS 39762, USA; Department of Biological Sciences, University of Memphis, Memphis, TN 38152, USA; Department of Wildlife, Fisheries, and Aquaculture, Mississippi State University, Starkville, MS 39762, USA; Department of Biological Sciences, University of Memphis, Memphis, TN 38152, USA; Department of Biochemistry, Molecular Biology, Entomology, and Plant Pathology, Mississippi State University, Starkville, MS 39762, USA

**Keywords:** Amphibian, eggs, hormone, ovulation, toad

## Abstract

Many amphibian species reinitiate the processes of preparing for reproduction (e.g. oogenesis) soon after breeding indicating hormone-induced ovulation could potentially be achieved out-of-season, which would lead to higher annual fecundity compared to mono-seasonal breeding. Such strategies would be beneficial to captive breeding programs for threatened species that are short-lived, have aging populations or need large numbers of offspring to meet reintroduction goals for species recovery. Unfortunately, little is known regarding how female anurans respond to multiple ovulation events within a year, which could lead to higher annual fecundity compared to mono-seasonal breeding. Thus, we evaluated the effect of temporal period between exogenous hormone stimulation events on egg production using the Fowler’s toad *Anaxyrus fowleri* as a model species. Female toads (*n* = 21) were administered hormone therapy twice in 1 year with toads randomly assigned to a treatment of either a 4-, 8- or 12-month recovery period between hormone stimulations. Ovulation was induced using two priming doses of human chorionic gonadotropin (100 IU; hCG) 72 h apart, followed by a resolving dose of hCG (500 IU) plus gonadotropin releasing hormone analogue (GnRHa; 15 μg) given 24 h after the second priming injection. Measured response variables include the number of females ovulating after treatment, total number of eggs produced and percent fertilization, neurula and tadpole development. No significant treatment effects were observed for any response variable (*P* > 0.05). Findings from this study suggest that hormone therapy can be administered in a *bufonid* species every 4 or 8 months without significantly affecting the number of ovulating females, egg production, fertilization, neurulation or tadpole development. By collecting gametes out-of-season or multiple times throughout the year, captive breeding programs could potentially increase tadpole production for reintroductions as well as extend the breeding window in captivity.

## Teaser

Several temperate toads undergo oogenesis soon after breeding which could be exploited by captive breeding programs to increase tadpole production. In this study, non-hibernated Fowler’s toads produced fertilizable oocytes after a four month refractory period without significantly impacting the number of ovulating females, total egg production, or egg quality.

## Introduction

An expanding number of amphibian species are experiencing population declines (40% threatened with extinction) (IUCN 2018), resulting in the establishment of captive-assurance colonies for several at-risk species to serve as a hedge against extinction (Gascon *et al.*, 2007; Harding *et al.*, 2016; Silla and Byrne, 2019). However, maintaining long-term founder lines and sustainable captive populations has been challenging due to poor reproductive output for a number of species (Gascon *et al.*, 2007; Kouba *et al.*, 2012b). Amphibian captive breeding can be problematic due to the difficulties of simulating environmental conditions necessary for natural reproduction causing breeding dysfunction such as failure to display reproductive behaviours, failure to produce eggs or sperm and asynchrony in egg/sperm release (Kouba *et al.*, 2009). Even recovery programs which have reintroduced captive bred offspring into the wild still struggle with consistently producing new offspring on a yearly basis (Browne *et al.*, 2006; Roth *et al.*, 2010). These challenges associated with long-term sustainability of genotypic variation and reproduction have hampered recovery goals set forth by many federal and state recovery plans (Silla and Byrne, 2019; Silla *et al.*, 2019).

Captive management plans have been able to reduce issues related to low reproductive output by incorporating assisted reproductive technologies (ART) to aid in propagation efforts (Kouba *et al.*, 2009; Bronson and Vance, 2019). For instance, Drietz (2006) states the Wyoming toad *Anaxyrus baxteri* has rarely bred in captivity without hormone stimulation administered to at least one sex and implementing exogenous hormone therapy had yielded > 100 000 tadpoles released back into the wild. ART aids captive breeding through the manipulation of amphibian reproductive processes, specifically steroidogenesis and gametogenesis (Kouba *et al.*, 2009). Exogenous hormones, such as human chorionic gonadotropin (hCG) and gonadotropin releasing hormone analogue (GnRHa), are often utilized in breeding programs to stimulate reproductive behaviours (e.g. calling, amplexus), ovulation or spermiation (Kouba *et al.*, 2012b; Clulow *et al.*, 2014; Bronson and Vance, 2019; Silla and Byrne, 2019).

Breeding in temperate amphibian species often occurs in a brief seasonal manner with development and activity of the male and female gonads related to environmental changes (Duellman and Trueb, 1986). Thus, recovery programs breeding at-risk species must coordinate efforts during a narrow 4–6-week window in order to stock tadpoles in the wild when resources are sufficient for individuals to reach a target size before hibernation (McDonough *et al.*, 2016). To prepare for this narrow breeding season, holding institutions will simulate hibernation for temperate species in captivity as it is considered a requirement for collecting fertilizable eggs or inducing reproductive behaviour (Roth *et al.*, 2010; Calatayud *et al.*, 2015). However, vitellogenic growth of oocytes is complete prior to hibernation in nature (Jørgensen, 1992) suggesting a potential to circumvent hibernation requirements with ART. Specifically, using ART combined with strategic timing of a secondary hormone stimulation attempt could reduce seasonal breeding issues by inducing ovulation in females outside of the small breeding window. This would allow recovery programs to extend the breeding season or collect gametes out-of-season in captivity (Kouba and Vance, 2009). Since breeding programs are tasked with producing as many tadpoles as possible for reintroduction and recovery purposes, the ability to collect gametes throughout the year may benefit captive breeding efforts by increasing tadpole production. For example, increasing tadpole production out-of-season could be used to head start animals for release for temperate anuran species or tadpoles could be reintroduced immediately in the case of tropical species.

Several studies have reported that anuran species prepare for reproduction quickly after breeding (Roth *et al.*, 2010) including oogenesis beginning soon after ovulation in the desert spadefoot *Scaphiopus couchii* (Harvey *et al.*, 1997) and Houston toads *Bufo houstonensis* (observed via ultrasound 4–6 weeks after ovulation; Kouba, unpublished data). Moreover, European toads *Bufo bufo* initiate vitellogenic growth within a few weeks of spawning in captivity (Jørgensen, 1992), and a large portion of the ovarian mass is restored within 1 month of post-oviposition in the American toad *Anaxyrus americanus* (Johnson *et al.*, 2002). Further, Trudeau et al. (2013) successfully bred northern leopard frogs *Lithobates pipiens* out-of-season using exogenous hormone stimulation (gonadotropin releasing hormone + metoclopramide), although authors utilized wild-caught frogs which were in hibernation when captured (mid-November through December). The effects of multiple reproductive events and the subsequent time allotment between hormone-induced ovulation attempts are not well studied in captive female anurans.

The collection of ovulated fertilizable eggs is often the rate-limiting step for ART development (Kouba et al. 2009), and understanding the effects of the duration between hormone stimulation attempts on egg production will help establish, and refine, captive breeding protocols. Therefore, the purpose of this study was to evaluate the effects of different temporal periods between hormone-induced ovulation on egg recruitment in female Fowler’s toad. Findings from this study will be beneficial to threatened and endangered amphibian breeding programs by evaluating different recovery periods between reproductive events in order to maximize egg production using the common Fowler’s toad as a model species.

## Methods

### Study species

*Anaxyrus fowleri* is a small bodied (5.1–7.5 cm) anuran that is commonly found across the eastern USA (Greenberg and Green, 2013; Jones and Tupper, 2015). *A. fowleri* can be found in wooded areas, river valleys, floodplains, agricultural areas and typically breeds in spring to early summer (Jones and Tupper, 2015). Breeding sites are usually associated with permanent or temporary water bodies including ditches, flooded fields and along shallows of lakes and slow-moving rivers (Vogel and Pechmann, 2010). Their conservation status is listed as least concern (IUCN 2018).

Fowler’s toads (*n* = 21; weight = 40 ± 2 g) were collected in the spring of 2009 and 2010 (April–May) in Shelby County, TN, during their natural breeding season under the Tennessee Wildlife Resources Agency Permit #3506. Toads were marked with a passive integrative transponder (PIT) tag (Biomark, Boise, ID, USA) to allow for individual identification. Toads were kept indoors in either tanks (178 × 81 × 36 cm; Waterlandtubs, Orange, CA, USA) or containers (61 × 40 × 31 cm, Rubbermaid, Fairlawn, OH, USA) stocked at 8–10 or 4–6 toads per unit, respectively. All tanks and containers provided toads with access to cover, a water reservoir and dried sphagnum moss. Toads were fed an alternating diet between crickets and mealworms twice a week. Experiments were conducted from August 2010 through August 2011. Toads were not hibernated, yet were exposed to a natural photoperiod (latitude = 35.1495° N, longitude = 90.0490° W) through a skylight and kept at a constant temperature (25°C) throughout the experimental period. Further, the water source for all fertilization and development trials came from the same aged tap water held at room temperature and all subsequent embryos produced were reared under identical conditions (e.g. similar water source, temperature, depth, lighting, feeding regimen) and separately from the adults. Husbandry and experimental protocols were reviewed and approved by the Memphis Zoo Institutional Animal Care and Use Committee (# 2008-09).

### Experimental design

This experiment follows a completely randomized design with individual female Fowler’s toads defined as the experimental unit. Female toads were randomly assigned to one of three treatments (*n* = 7 toads per treatment), which were designed to evaluate the effects of different temporal periods between hormone-induced ovulation attempts on egg production and recruitment. Toads were subjected to two hormone-induced ovulation events with either a 4-, 8- or 12-month recovery period between the first and second hormone regimen (Table 1).

**Table 1:** Mean weight ± standard error for each treatment at the time of the first hormone stimulation as well as the timeline for the first and second hormone stimulations for each treatment

**Treatment**	**Initial stimulation**	**Second stimulation**
4 months	Aug. 2010	Dec. 2010
8 months	Aug. 2010	May 2011
12 months	Aug. 2010	Aug. 2011

All toads were given the first hormone stimulation in August 2010 to expel existing oocytes. Ovulation was induced using a protocol of two priming injections followed by a resolving injection. Priming doses were spaced 72 h apart and consisted of 100 IU hCG (Product #C-1063; Sigma-Aldrich, St. Louis, MO, USA) in 200 μL saline each. The resolving, or ovulatory, dose was given 24 h after the final priming injection and consisted of 500 IU hCG + 15 μg GnRHa (Product # L4513; LHRHa; Sigma-Aldrich, St. Louis, MO, USA). All injections were administered in the intraperitoneal cavity as described in Kouba et al. (2012a). Following injections, toads were placed in individual plastic containers (37 × 21 × 12 cm; Sterilite, Townsend, MA, USA) with approximately 1 cm of water to encourage water absorption important for egg jelly production. Toads were allowed to spontaneously ovulate for 24 h following the resolving dose with the number of ovulated eggs counted and recorded for each female. This hormone stimulation protocol was then repeated with the same female toads after the assigned treatment period except eggs collected in the second hormone stimulation were also fertilized to evaluate egg quality.

### IVF protocol and spermic urine collections

For the second ovulatory hormone stimulation event, all expelled eggs were collected and counted, with approximately 100–200 eggs expressed into individual petri dishes for fertilization. The reason for the variation in eggs expelled per female was due to rapid expulsion of eggs and an attempt to minimize auto-activation due to manipulation. Although the number of eggs taken to fertilization differed by treatment (500–1000 per female), we are quantitatively measuring the ratio of fertilized to non-fertilized eggs to provide a percent fertilization with a minimum of 500 eggs/female to make meaningful assumptions. A minimum of three males were used for fertilizations of eggs to mitigate any potential male or sperm quality effects on fertilization trials with spermic urine samples from the three males being pooled together, thoroughly mixed and diluted to a standardized concentration of 1 × 10^6^ spermatozoa per millilitre using previously collected, non-spermic toad urine as the diluent. Work in our lab has shown that this sperm concentration for 100–500 eggs is at the plateau of the fertilization curve and establishes an appropriate sperm:egg ratio for comparing treatments. Fertilization was initiated by gently pipetting 100 μL of spermic urine at the standardized concentration onto 100–200 eggs per petri dish and letting the resulting sperm/egg mixture sit for 5 min before flooding the petri dish with aged tap water.

To obtain spermic urine for IVF, spermiation was induced in 10 male Fowler’s toads using a hormone injection of 300 IU hCG in 100 μL saline. Following hormone injections, toads were placed in a plastic container with water similar to the container described previously. Spermic urine samples were collected 3–6 h after the hormone injection by removing toads from the container, quickly drying excess water from the abdomen and holding toads over a 15-cm petri dish to collect urine. Samples were immediately evaluated for motility, quality of forward progressive movement (QFPM) and concentration following collection. Motility was calculated by conducting a random count of 100 spermatozoa and classifying each as motile or non-motile. Motile spermatozoa was defined by having a moving flagellum regardless if forward movement was observed or not. Forward movement was scored on a scale of 0 (no movement) to 5 (rapid forward movement) to quantify quality of FPM. This QFPM scale has been used extensively in amphibian research previously (Obringer *et al.*, 2000; Rowson *et al.*, 2001; Browne *et al.*, 2006; Kouba *et al.*, 2012a; Kouba *et al.*, 2012b; McDonough *et al.*, 2016). Spermatozoa concentration (sperm/mL) was measured on a hemacytometer using a 1:10 diluted sample in saline. Only samples with a ≥90% motility, ≥3 FPM and ≥${10}^6$ spermatozoa concentration were utilized for IVF. Samples were stored in a refrigerator (${4}^{\mathrm{o}}\mathrm{C}$ for < 24 h) and reassessed for the same sperm quality standards before being used to fertilize eggs.

**Figure 1:**
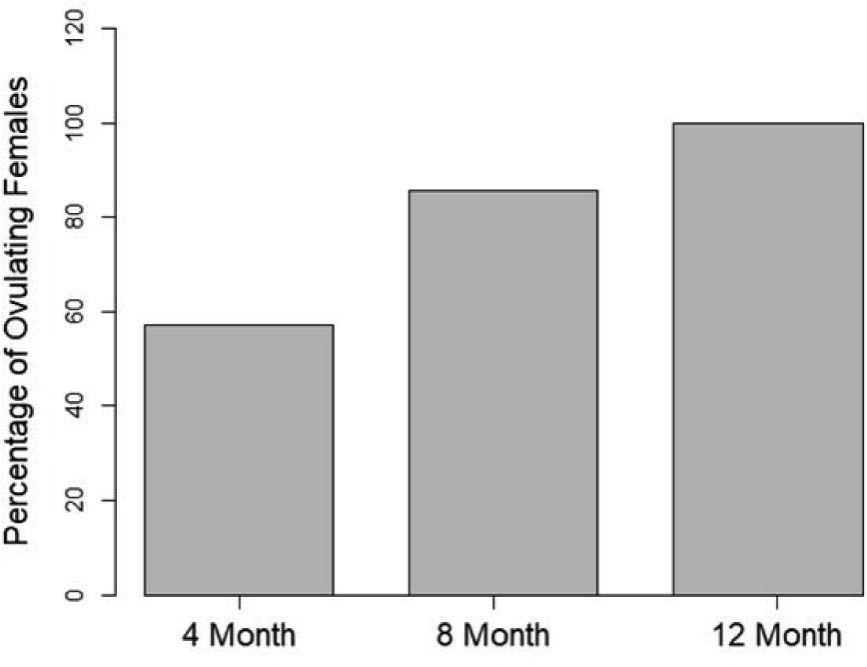
Percentage of females (*n* = 7/treatment) which responded to the second hormone stimulation at 4 months (*n* = 4), 8 months (*n* = 6) and 12 months (*n* = 7) after the first resolving hormone stimulation

Stages of fertilization and embryonic development were then recorded and quantified using methods similar to Browne et al. (2006). Fertilization percentage was calculated by counting the number of cleaved eggs (larval stage 3–6; recorded 4–6 h after sperm was applied to eggs; Nieuwkoop and Faber, 1967) and unfertilized eggs for each petri dish. Similarly, neurulation percentage was determined by counting the number of eggs in the neurula stage of development (larval stage 26–28; Nieuwkoop and Faber, 1967) while tadpole percentage was calculated by counting the amount of embryos reaching swim-up tadpole stage (larval stage 44–46; Nieuwkoop and Faber, 1967). Count data for the individual petri dishes were then added together to obtain a total amount for each female in order to evaluate the following variables: fertilization percentage, neurula percentage and tadpole percentage.

**Figure 2:**
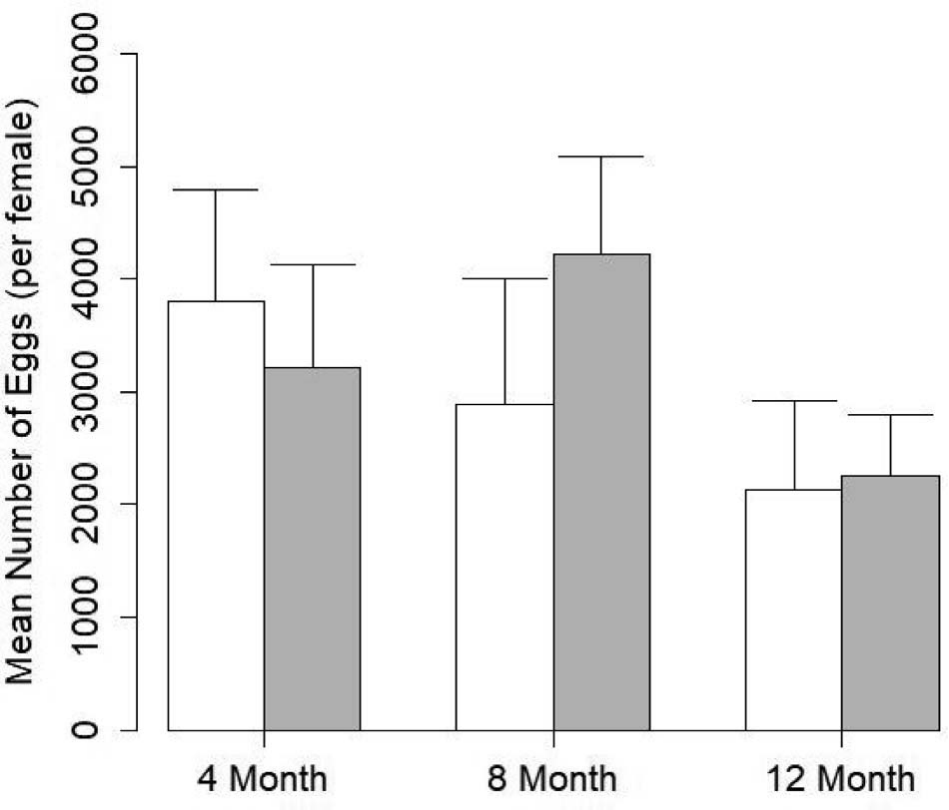
Mean number of eggs produced before and after the assigned temporal period. Mean (+ SEM) number of eggs produced per female for the 4-month (*n* = 4), 8-month (*n* = 6) and 12-month (*n* = 7) treatment. White bars represent mean amount of eggs produced in the first hormone stimulation while shaded bars represent the amount of eggs produced in the second hormone stimulation. Mean number of eggs produced per female was compared to the amount of eggs produced by the same toads after the initial hormone stimulation with a paired *t* test. No significant difference was observed between the first and second hormone stimulation for any treatment group (*P* < 0.05)

### Statistical analysis

The number of female toads ovulating in each treatment was analyzed with a Fisher’s exact test. Responding females were defined as those that ovulated and were assigned a value of 1 while non-responding females were defined as females which did not ovulate and were assigned a value of 0. The resulting binary data were compiled into a 2 (observed binary responses) by 3 (treatments) contingency table prior to analysis.

Comparisons between the mean number of eggs produced during the first hormone stimulation and the hormone stimulation after the assigned temporal period were analyzed using a paired *t* test. Since we were interested in evaluating differences in egg production in the females that responded to both hormone stimulations, only females which ovulated at both hormone stimulations were included in the analysis.

The clutch size (total amount of eggs ovulated) between treatments, fertilization percentage from total eggs ovulated, neurula percentage from total eggs ovulated and tadpole percentage from total eggs ovulated were analyzed using an analysis of covariance (ANCOVA) where the fixed effect factor was the assigned treatment temporal period between the first and second hormone stimulation, while weight (at time of second stimulation) was controlled for as a covariate. Female toads which did not ovulate at the second hormone stimulation were omitted from the analysis due to the inability to fertilize non-existent eggs. Differences between treatment means were considered significant at *P* < 0.05 with a Tukey’s multiple comparison test used for pairwise comparisons when significant treatment effects were detected. No significant interaction effects between treatment and female weight was not observed for any response variable, and thus, the interaction term was removed from the model statement.

**Table 2:** Mean ± standard error results of egg quality variables from the 4-, 8- and 12-month treatments

**Variable**	**Treatments**			
	**4 months**	**8 months**	**12 months**	***P* value**
*N*	4	6	7	**-**
Total eggs released after treatment	12 868	25 322	15 785	**-**
Average clutch size per female	3217 ± 919	4220 ± 866	2255 ± 545	0.10
Eggs attempted to fertilize (per female)	572 ± 73	758 ± 81	1082 ± 107	**-**
% Fertilized	48.5 ± 11.4	61.0 ± 7.8	52.0 ± 9.9	0.63
% Neurulas	35.5 ± 11.0	50.5 ± 9.3	26.4 ± 7.4	0.15
% Tadpoles	32.1 ± 10.0	46.6 ± 9.2	18.6 ± 8.0	0.1

The percent fertilized eggs, eggs reaching neurula stage and tadpole percentage are calculated based off the total number of eggs attempted to fertilize per female. Average clutch size was analyzed between treatments. Different lowercase letters represent significance across rows (α < 0.05).

To examine the body condition of the animals during the experiment and changes between hormone stimulations, we analyzed the initial weight, final weight and change in weight over the experimental period (i.e. weight at first hormone stimulation minus weight at second hormone stimulation) using an analysis of variance (ANOVA) where the fixed effect factor was the assigned temporal period. Differences between treatment means were considered significant at *P* < 0.05 with a Tukey’s multiple comparison test used for pairwise comparisons when significant treatment effects were detected.

Assumptions of normality and homogeneity of variance were checked using the Shapiro-Wilk and Levene’s test, respectively. Percentage data were transformed using an arcsin-square-root transformation before analysis. Statistical analysis was conducted using R Core Team (R Core, 2018), and differences between means were considered significant at *P* < 0.05.

## Results

The temporal period between hormone stimulations did not have a significant effect (*P* = 0.29) on the amount of females responding to the second hormone stimulation (Fig. 1). Four toads (57%) ovulated in the 4-month treatment. Six toads (86%) ovulated in the 8-month treatment, while all seven toads (100%) ovulated in the 12-month treatment.

The mean number of eggs produced between the first and second hormone stimulations was not significantly different regardless of temporal period between hormone stimulation (Fig. 2). Specifically, the average number of eggs produced in the first hormone stimulation did not differ significantly from the number of eggs produced in the 4 month (Mean ± SE = 3799 ± 997 vs 3217 ± 919; *P* = 0.73), 8 month (2892 ± 1111 vs. 4220 ± 866; *P* = 0.20), or 12-month treatment (2136 ± 782 vs 2255 ± 545; *P* = 0.92).

No significant effects of temporal period between hormone stimulations were observed for clutch size when analyzed between treatments (*P* = 0.10), fertilization percentage from total eggs ovulated (*P* = 0.64), neurula percentage from total eggs ovulated (*P* = 0.16) or tadpole percentage from total eggs ovulated (*P* = 0.10) (Table 2). Weight at time of second hormone stimulation did not significantly affect fertilization rates or embryonic development variables. Lastly, the initial weight of female Fowler’s toads (*P* = 0.26), the final weight (*P* = 0.57) and the change in weight over the experimental period (*P* = 0.42) did not differ between the treatments (Table 3).

**Table 3:** Mean ± standard error weight results for female Fowler’s toads in the 4-, 8- and 12-month treatments

**Treatment**	***N***	**Initial weight (g)**	**Weight at second treatment (g)**	**Change in weight (g)**
4 months	7	42 ± 4	41 ± 4	1 ± 2
8 months	7	39 ± 4	42 ± 4	−3 ± 2
12 months	7	38 ± 3	40 ± 3	−2 ± 2
*P* value	-	0.26	0.57	0.42

## Discussion

This study provides a clearer understanding of oocyte recruitment and maturation in amphibians, which could be exploited for increasing reproductive output, sustainability and head-starting tadpole production for threatened species in captive breeding programs. Although amphibians undergo a natural refractory period after breeding (Duellman and Trueb, 1986), vitellogenic growth is completed prior to hibernation in the wild in temperate species (Jørgensen, 1992) indicating that the process of hibernation is not necessary for initial oocyte maturation. Findings in this study demonstrate oocyte recruitment occurs within 4 months post-oviposition in non-hibernated Fowler’s toads. With other studies reporting oogenesis occurring soon after ovulation in several species, a strategy of collecting gametes out-of-season could potentially be utilized by breeding programs to produce tadpoles out-of-season or repeatedly throughout the year. Results from this study provide insight into such a strategy by suggesting two hormone stimulations can be administered 4 or 8 months apart without a significant effect on the number of ovulating females, total number of ovulated eggs or egg quality in a *bufonid* species.

The reasoning for amphibian species to begin the energetic process of preparing for reproduction soon after breeding is not readily apparent. Vitellogenic growth of oocytes is a costly process for individuals, particularly after resources were spent during the breeding season. In fact, ovarian quiescence in spring-breeding anurans is thought to be adaptive, ensuring oocyte growth (vitellogenesis) is postponed until the energy reserves of the animal are restored (Jørgensen, 1992). Findings in this study indicate that oocyte recruitment, vitellogenesis and initial maturation occur within 4 months in non-hibernated Fowler’s toads and exogenous hormone applications were able to exploit this quick oocyte production by collecting fertilizable eggs out-of-season. With the effectiveness of ART being dependent upon exerting control over the timing of ovarian reproductive cycles, capitalizing on the quick reproductive process displayed in some amphibian species could prove beneficial to recovery programs considering results from this study also suggest egg quality variables did not differ between treatments. This strategy of producing offspring out-of-season would be effective for recovery programs that need large numbers of tadpoles for reintroduction goals while also providing breeding programs the opportunity to head-start tadpoles for release at larger juvenile sizes during the summer months. Additionally, the need for simulating hibernation in captivity could potentially be bypassed by adopting a captive breeding strategy which breeds temperate toads out-of-season.

Hibernation is thought to be an important stimulus for inducing reproductive behaviour or collecting fertilizable eggs in female temperate toads (Reading, 1998; Bronson and Vance, 2019). During hibernation in nature, the function of the toad ovary switches from vitellogenic growth to follicular maturation which eventually leads to the completion of a normal ovarian cycle after hibernation through the rupture of mature follicles and extrusion of oocytes into the body cavity (Jørgensen, 1992). Thus, it is understandable why exposing toads to low temperatures during hibernation is believed to be important in controlling their annual ovarian cycle and inducing reproductive behaviour. However, we were able to collect fertilizable eggs from wild-caught individuals which were kept out of hibernation and maintained in captivity for up to 1 year. Roth *et al.* (2010) observed similar results where authors collected, and successfully fertilized, eggs from non-hibernated Boreal toads *Anaxyrus boreas boreas*, although hibernation in combination with exogenous hormones still appears to be helpful for effectively inducing ovulation in this species (Calatayud *et al.*, 2015). It is not clear what role hibernation plays in preparing females for reproduction in captive populations, which has led to researchers evaluating whether hibernation requirements can be bypassed and ovulation successfully induced in non-hibernated individuals using exogenous hormones (Roth *et al.*, 2010; Calatayud *et al.*, 2015). The ability to induce ovulation in non-hibernated Fowler’s toads in this study could be partially due to toads originating from a mild climate (Southeastern United States) and, thus, are adapted to completing ovarian cycles in the absence of cold temperatures and hibernation. It is unknown if Fowler’s toads require a certain level of hibernation in natural breeding, although we have not observed any captive toads initiate calling, amplexus or spawning under captive conditions in which hibernation was not undertaken. While more experiments are needed to test the necessity of hibernation for developing species-specific protocols, this study demonstrates that proper reproductive processes occur in captive, non-hibernated Fowler’s toads and similar fertilization levels can be achieved compared to hibernated, wild-caught Fowler’s toads.

Like many of the decisions managers face, the benefits gained from implementing a change in a captive breeding protocol must be balanced with equal consideration of the negative impacts the change could have on the animal’s health. For instance, inducing ovulation too frequently could result in adverse effects such as weight loss and decreased reproductive output, particularly considering that oocyte production is an energetically costly process. Repeated hormone therapy is speculated to have played a role in observed decreases in body weight and sperm production in male Fowler’s toads treated with hormones 2× per week (McDonough *et al.*, 2016). Changes in body weight between hormone stimulations remained consistent across the treatments used in this study, despite toads in the 4-month treatment subjected to a shorter recovery period. These results indicate that 4 months is an adequate amount of time for female Fowler’s toads to replenish energy reserves in captive conditions. Further, toads in all treatments produced similar amounts of eggs when compared to the initial induced ovulation event after capture from the wild, which also suggests an expedited recovery period of 4 months does not harm short-term (<1 year) reproductive health. Since evaluating long-term (>1 year) repercussions of exposing female anurans to repeated hormone stimulation was beyond the scope of this experiment, future studies could investigate the effects multiple ovulation events in a year have on the long-term health of captive female anurans (e.g. life expectancy, egg output from year to year).

Interestingly, the temporal period between hormone stimulations used in this study had no statistical effect on the number of ovulating females during the second hormone stimulation despite a wide margin between the 4- and 12-month treatments. One possible reason for this margin is the lower sample size (21 animals split between 3 treatments), and given additional animals, the significance may have become more apparent. Moreover, the lower response rate in the 4-month treatment could have been impacted by a seasonality effect meaning environmental stimuli that are present in August were likely not present in December. Amphibians rely on environmental cues to stimulate reproduction (Duellman and Trueb, 1986), and certain stimuli are difficult to re-create in the lab (Kouba and Vance, 2009; Bronson and Vance, 2019). Despite major parameters that stimulate reproductive behaviours (temperature, food availability, etc.) being held constant throughout the experiment, minor cues (e.g. barometric pressure) could not be maintained consistently, which might have aided in the observed response rate. It is worth noting that the total number of ovulated eggs as well as egg quality variables were similar, regardless of when the second hormone stimulation was received. Future experiments could focus on further evaluating the seasonality timing of the two hormone stimulations given 4 months apart (i.e. April–August, August–December or December–April) and the corresponding effects on the number of ovulating females.

ART has the potential to enhance propagation and reintroduction efforts as inducing reproductive behaviour and gamete collection is often a limiting factor for captive breeding programs (Kouba and Vance, 2009). This study adds to the existing literature by evaluating different temporal periods between hormone-induced ovulation events and the resulting effects on the number of ovulating females and egg production. Overall, results from this study indicate that two hormone stimulations can be administered 4 or 8 months apart in the Fowler’s toad without significantly impacting the number of ovulating females, overall egg production or egg quality parameters (fertilization percentage, neurulation percentage or tadpole percentage). Further, results in this study demonstrate that reproductive processes, such as egg recruitment, occur soon after breeding (<4 months) which is consistent with other studies. Therefore, breeding programs for several threatened amphibian species may be able to take advantage of oogenesis occurring quickly after ovulation, by collecting gametes out-of-season or multiple times in a year and, thus, providing recovery programs the ability to increase production output. With amphibian species experiencing population declines and issues with captive breeding threatening the long-term success of recovery programs (Kouba and Vance, 2009), studies evaluating ART methods are valuable and will continue to aid conservation efforts.

## Funding

This work was supported by the Institute of Museum and Library Services National Leadership Grant (LG-25-09-0064-09) and Morris Animal Foundation (D09ZO-032).
